# Structural and cultural barriers to patient education from the perspective of Iranian nurses: a qualitative study

**DOI:** 10.1186/s12912-026-04819-1

**Published:** 2026-06-01

**Authors:** Safura Yaghmaei, Nazanin Rajabi Kahrizi, Zahra Ranjipour, Shiva Gomarverdi, Fariba Tayebi Arasteh, Zeinab Mosivand, Roya Ghahremani

**Affiliations:** 1 Department of Psychiatric Nursing, School of Nursing and Midwifery, Hamadan University of Medical Science, Hamadan, Iran; 2 Hamadan University of Medical Sciences, Hamadan, Iran; 3 Clinical Research Development Unit Farshchian Heart Center Iran, Hamadan University Medical Science, Hamadan, Iran; 4 Clinical Research Development Unit Farshchian Sina Center Iran, Hamadan University Medical Science, Hamadan, Iran; 5 Clinical Research Development Unit Besat Hospital, Hamadan University Medical Science, Hamadan, Iran; 6 Clinical Research Development Unit Fatemieh Hospital, Hamadan University Medical Science, Hamadan, Iran; 7 Clinical Research Development Unit Beheshti Hospital, Hamadan University Medical Science, Hamadan, Iran

**Keywords:** Patient education, Nursing, Cultural barriers, Structural barriers, Qualitative research, Iran, Hospital policy

## Abstract

**Aim:**

This study examines the structural and cultural barriers that hinder patient education (PE) among nurses in Iranian public hospitals, focusing on the unique challenges in Hamadan’s healthcare system.

**Design:**

A qualitative study using conventional content analysis.

**Methods:**

We conducted semi-structured interviews with 11 nurses (8 female, 3 male) from five public hospitals in western Iran between September 2023 and July 2025. Participants, selected purposively for diverse clinical experience, shared their views on PE barriers. Interviews were transcribed verbatim and analyzed using Graneheim and Lundman’s content analysis approach.

**Results:**

Nurses identified four key barriers: organizational limitations, cultural resistance, patient-related challenges, and personal professional constraints. These barriers, captured under the theme “Culturally silenced care,” reveal how hospital policies, such as mandatory PE requirements, often undermine quality education, while cultural attitudes and limited resources further marginalize nurses’ educational roles.

**Conclusion:**

To strengthen PE in Iranian hospitals, targeted reforms are needed, including better resource allocation, cultural sensitivity training, and dedicated spaces for education. These insights can guide nursing practice in Iran and similar resource-limited, hierarchical healthcare systems worldwide.

**Supplementary Information:**

The online version contains supplementary material available at 10.1186/s12912-026-04819-1.

## Introduction

In real-world hospital settings, barriers to patient education (PE) are not abstract, they are lived. consider the following vignettes drawn from common hospital scenarios:

A young nurse in an overcrowded emergency unit notices that a newly admitted heart failure patient needs education on fluid restriction and medication adherence. Overwhelmed by discharge paperwork, staff shortages, and competing demands, she reluctantly walks away, unable to provide the education she knows is essential. In another instance, a nurse attempting to explain post-operative care is interrupted by family members who insist on managing things “their own way,” leading her to withdraw to avoid cultural conflict.

These snapshots illustrate how structural constraints, cultural resistance, and intra-professional norms work in tandem to silence nurses’ educational roles. These scenarios are not isolated incidents but reflections of a broader and persistent issue in hospital nursing: the marginalization of patient education (PE), particularly in resource-limited and culturally complex contexts.

Patient education (PE) is a vital part of nursing, helping patients manage their health and make informed choices. Yet, in the busy wards of Iranian public hospitals, nurses often struggle to provide this essential care. Picture a young nurse in a crowded emergency unit in Hamadan, trying to explain medication use to a heart failure patient but swamped by paperwork and short-staffed shifts. In another ward, a nurse starts discussing post-surgical care, only to face interruptions from a patient’s family who prefer their traditional methods. These real challenges show how hospital systems, cultural beliefs, and workplace pressures often prevent nurses from fulfilling their educational roles.

As a cornerstone of patient-centered care, PE empowers individuals to take charge of their health, reduces anxiety, and improves outcomes, such as fewer hospital readmissions [1, 2]. The World Health Organization describes PE as an ongoing process that provides tailored information to support self-care and better health [3]. For nurses, PE is both an ethical duty and a legal responsibility, contributing to better patient satisfaction and lower healthcare costs [4, 5]. However, delivering effective PE is not easy, especially in resource-limited settings where nurses face significant barriers [6].

In Iran’s public hospitals, nurses encounter unique challenges that limit PE. Heavy workloads, insufficient staffing, and a lack of dedicated spaces for education often push PE to the sidelines [7, 9]. Cultural factors, such as patients’ reliance on family advice or misconceptions about nurses’ roles, add further complexity [10]. In Hamadan, a region with diverse healthcare needs and limited resources, these issues are especially pronounced, yet few studies have explored nurses’ perspectives in this context [11]. While prior research in Iran has highlighted barriers like time constraints or lack of skills [7, 8], the combined impact of hospital policies and cultural dynamics remains underexplored [12].

Hamadan province, with its mix of urban and semi-rural public hospitals serving a culturally diverse population under resource constraints, provides a relevant context for exploring these challenges. While previous Iranian studies have examined barriers to PE, primarily focusing on time constraints, knowledge deficits, or patient-related factors, few have specifically addressed the interplay of structural and cultural barriers from nurses’ perspectives in this setting, nor have they systematically applied a quality-of-care framework to analyze them.

This study fills this gap by examining the structural and cultural barriers to PE from the perspectives of nurses in Hamadan’s public hospitals. Using Donabedian’s framework of healthcare quality (structure, process, outcome) [13], we explain how structural elements (e.g., staffing, resources, policies), process-related factors (e.g., workload, time, interpersonal dynamics), and their resulting outcomes interact to limit nurses’ educational roles. we explore how these barriers interact to limit nurses’ ability to educate patients. Our findings aim to inform practical solutions for improving PE in Iran and other resource-limited, hierarchical healthcare systems worldwide.

## Methods

### Design

This qualitative study employed conventional content analysis [14] to explore structural and cultural barriers to patient education from the perspectives of nurses. Data collection was conducted from September 2023 to July 2025 in five public hospitals in Hamadan, western Iran. The extended study period allowed for prolonged engagement with the research setting, seasonal variations in hospital workload, and iterative data collection and preliminary analysis.

### Setting and participants

The study was carried out in five public teaching hospitals affiliated with Hamadan University of Medical Sciences. Participants were purposively sampled to achieve maximum variation in terms of age, gender, years of clinical experience (2–30 years), ward type (internal medicine, surgery, emergency, ICU, and others), and educational level.

Inclusion criteria were: (1) registered nurses currently working in the study hospitals, (2) at least six months of clinical experience, and (3) willingness to participate. Exclusion criteria included unwillingness to participate or having less than six months of hospital experience.

Data saturation was monitored prospectively. No new codes or categories emerged after the eighth interview, and the final three interviews were conducted to confirm saturation. sample size is consistent with similar qualitative studies exploring nurses’ experiences in Iran and other middle-income countries.

### Data collection

Data were collected through semi-structured, in-depth interviews conducted by the first author, A university faculty member with a PhD in nursing, trained in qualitative methods. An interview guide, developed based on prior literature [6], included open-ended questions such as: “Can you describe your experiences with providing PE in your daily practice?”; “What factors prevent effective PE during your shifts?”; and “How do hospital policies or cultural factors influence PE delivery?” Probing questions (e.g., “Can you elaborate on how patient attitudes affect PE?”) were used to deepen responses. Interviews took place in private break rooms after participants’ shifts, lasting 37–70 min. All interviews were audio-recorded with participants’ consent and transcribed verbatim. To minimize interviewer bias, the first author maintained a reflexive journal, and preliminary interviews were discussed with the research team.

### Data analysis

Data were analyzed using Graneheim and Lundman’s conventional content analysis approach [14]. The five authors independently transcribed and reviewed interviews to identify key ideas. Each transcript served as a unit of analysis, with meaning units (words, sentences, or paragraphs) identified and condensed. These were coded based on latent meanings, grouped into subcategories and categories through iterative discussions, and synthesized into an overarching theme. For example, the quote “Mandatory PE feels like a checkbox exercise” was coded as “superficial compliance,” contributing to the subcategory “policy-driven barriers” and the category “organizational-managerial constraints.” Regular team meetings ensured consensus on coding and categorization. To enhance transparency of the analytic process, an example of the coding trajectory (from meaning unit to final theme) is provided in Tables 1 and 2. Code frequencies were calculated internally to identify the relative dominance of each category but were not used to quantify the findings. The final Table 3 presents only the categories, subcategories, and representative quotations.

**Table 1:** Analytical matrix of barriers to patient education

Main Category	Subcategory	Code Frequency
Organizational and Managerial Barriers	Low organizational priority of PE	38
Organizational and Managerial Barriers	Inappropriate methods and space	21
Organizational and Managerial Barriers	Ineffective workforce management	29
Organizational and Managerial Barriers	Inefficient reward systems & mandatory PE	24
Organizational and Managerial Barriers	Complex processes and non-nursing roles	19
Cultural Resistance	Clients’ non-acceptance of PE	17
Cultural Resistance	Nurses’ negative attitudes toward PE	20
Cultural Resistance	Perceived ineffectiveness of PE	15
Cultural Resistance	Misinterpretation of nurse-patient relationships	18
Cultural Resistance	Perceived difficulty of PE	14
Patient-related Barriers	Patients’ and companions’ conditions	30
Patient-related Barriers	Educational level and social class	22
Patient-related Barriers	Communication-related barriers	16
Patient-related Barriers	Mental concerns	18
Patient-related Barriers	Disease type	11
Nurse-related Barriers	Low motivation and emotional exhaustion	25
Nurse-related Barriers	Lack of PE-related knowledge/skills	27
Nurse-related Barriers	Task prioritization over PE	23
Nurse-related Barriers	Role ambiguity	20

**Table 2:** Example of the coding trajectory from meaning unit to overarching theme

Meaning Unit (Raw Data)	Condensed Meaning Unit	Code	Subcategory	Category	Overarching Theme
“We have to do patient education because the hospital requires it, but honestly, it’s just a checkbox. Nobody really cares about the outcome.” (P2)	Mandatory PE is done only for documentation and checking a box, not for real patient benefit.	Superficial compliance / Checkbox mentality	Inefficient reward systems and mandatory PE requirements	Organizational-Managerial Barriers	Culturally Silenced Care
“Patients don’t listen to us. They say ‘the doctor knows better’ and wait for the physician to explain everything.” (P5)	Patients reject nurse-led education due to belief in physician superiority.	Physician-centric culture / Lack of acceptance of nurse as educator	Lack of patient acceptance of education	Cultural Resistance	Culturally Silenced Care
“With 20 patients in the ward and only two nurses, I don’t even have time to explain simple things like how to take medicine.” (P7)	Extreme workload and poor nurse-patient ratio leaves no time for education.	High workload and inadequate staffing	Ineffective workforce management	Organizational-Managerial Barriers	Culturally Silenced Care
“I get so tired and burned out after the shift that I don’t have any energy left to teach patients properly.” (P10)	Emotional exhaustion and burnout reduce motivation for patient education.	Burnout and low motivation	Low motivation and emotional exhaustion	Nurse-Related Barriers	Culturally Silenced Care
“Some patients speak Turkish or Kurdish dialect and I can’t communicate well with them. Education becomes useless.” (P6)	Language and dialect differences between nurse and patient hinder effective education.	Linguistic and communication barriers	Language and communication limitations	Patient-Related Barriers	Culturally Silenced Care

**Table 3:** The subcategories and categories of the barriers to patient education

Theme	Category	Subcategories
Culturally Silenced Care	Organizational-Managerial Barriers	- Low organizational priority of patient education - Inappropriate methods and space - Ineffective workforce management - Inefficient reward systems and mandatory PE requirements - Complex processes and excessive non-nursing roles
	Cultural Resistance	- Lack of patient acceptance of education - Nurses’ negative attitudes toward education - Belief in the ineffectiveness of education - Misunderstandings in nurse-patient communication - Considering education as difficult
	Patient-Related Barriers	- Patients’ and caregivers’ psychological and physical conditions - Low literacy level and socioeconomic status - Language and communication limitations - Mental preoccupations - Type of illness
	Nurse-Related Barriers	- Low motivation and emotional exhaustion - Lack of PE-related knowledge/skills - Inability to manage personal problems - Poor individual and social skills - Task prioritization over PE - Role ambiguity

### Rigor/trustworthiness

Rigor was ensured using Lincoln and Guba’s criteria: credibility, dependability, confirmability, transferability, and authenticity [15].

Trustworthiness was established using Lincoln and Guba’s (1985) criteria:

Credibility: Achieved through prolonged engagement (18 months of regular presence in the study hospitals by the first author, including informal observations and discussions with nurses), maximum variation sampling, peer debriefing (regular meetings with two experienced qualitative researchers), and member checking (findings were returned to three participants who confirmed the overall interpretation).

Dependability and Confirmability: Maintained via an audit trail (detailed documentation of all analytic decisions), reflexivity, and an external audit conducted by an independent qualitative researcher not involved in the study.

Transferability: Supported by thick description of the context, setting, and participant characteristics.

Authenticity: Ensured by using verbatim quotations and staying close to participants’ voices.

The study is reported in accordance with the Consolidated Criteria for Reporting Qualitative Research (COREQ) checklist.

### Ethical considerations

The study was approved by the Ethics Committee of Hamadan University of Medical Sciences, Hamadan, Iran (code: IR.UMSHA.REC.1401.908). Participants were informed of the study’s purpose, assured of data confidentiality, and notified of their right to withdraw without affecting their employment. Written informed consent for participation and audio-recording was obtained from all participants. All procedures performed in this study were in accordance with the ethical standards of the institutional research committee and the Declaration of Helsinki.

## Results

Data from 11 nurses (8 women, 3 men) from five public hospitals affiliated with Hamadan University of Medical Sciences, Iran, were analyzed, yielding 304 primary codes, condensed into 217 non-overlapping codes, 21 subcategories, and four main categories (Table 3). Participants’ characteristics, including age (range: 24–49 years, mean: 35.25), work experience (range: 2–25 years, mean: 12), and ward diversity (e.g., pediatric, coronary care, emergency, oncology, neurology), are presented in Table 4. To ensure full participant anonymity, Age was converted from exact numerical values to broader age ranges (e.g., 25–30) and Gender information was removed, as the combination of gender + age + years of experience + workplace could potentially identify participants in specific hospital units. All other demographic data were reviewed to ensure no indirect identifiers remain.

**Table 4:** Participants’ characteristics

No	Age (Years)	Work experience (Years)	Affiliated ward	Interview duration (Minutes)
1	20_25	2	Pediatric	48.40
2	30_35	11	Coronary care	33.53
3	40_45	22	Medical	27.28
4	30_35	7	Emergency	38.50
5	40_45	22	Oncology	40.28
6	30_35	10	Orthopedic	50.20
7	30_35	8	Trauma	60.00
8	20_25	2	Neurology	38.20
9	35_40	15	Neonatal	40.30
10	45_50	25	Mental care	25.00
11	30_35	8	Urology	35.20

The categories, organizational-managerial constraints, cultural resistance, patient-related limitations, and nurse-related challenges, were synthesized under the theme “Culturally silenced care,” illustrating how systemic and sociocultural barriers interact to marginalize patient education (PE) in Iran’s public hospitals, reducing it to a superficial, often neglected task. Drawing on Donabedian’s framework of healthcare quality (structure, process, outcome) [13], these findings highlight how structural deficits, process-related barriers, and their resultant outcomes undermine nurses’ educational roles in Hamadan’s culturally complex, resource-limited context. Code frequencies, illustrating the prevalence of each barrier, are presented in Tables 4 and 1.

### Organizational-managerial constraints

Institutional barriers, rooted in Donabedian’s structural domain [13], significantly hindered PE by limiting resources and prioritizing clinical tasks over education. Nurses described how hospital systems devalued PE, creating a cycle of neglect and superficial compliance.

- **Low organizational priority of PE**: Nurses consistently reported that hospital leadership undervalued PE, lacking formal systems for its documentation or evaluation. “*PE isn’t prioritized; it’s overshadowed by tasks like infection control or charting*” said one nurse (P10, female, 25 years’ experience).
- **Inappropriate methods and space**: The lack of dedicated spaces and modern educational tools (e.g., videos, brochures) forced nurses to conduct PE in chaotic bedside settings. “*There’s no private room for teaching; we’re stuck educating at the bedside with interruptions*,” noted a nurse (P3, male, 22 years’ experience).
- **Ineffective workforce management**: High workloads and low nurse-patient ratios compelled nurses to prioritize urgent clinical tasks over PE. “*With 20 patients per shift*,* PE gets sidelined; I barely have time to check vitals*,*”* shared a nurse (P7, male, 8 years’ experience). This issue was compounded by frequent staff shortages, a common structural deficit in Iran’s public sector .
- **Inefficient reward systems and mandatory PE requirements**: Mandatory PE policies, intended to ensure accountability, paradoxically reduced quality by fostering superficial compliance. “*We do PE to check a box*,* not because it’s valued*,” explained a nurse (P2, male, 11 years’ experience). This “*checkbox exercise”* led to rushed, ineffective sessions, undermining patient outcomes .
- **Complex processes and excessive non-nursing roles**: Administrative tasks, such as excessive paperwork and coordination with other departments, consumed nurses’ time. “*Paperwork and coordination eat up our energy*; *PE becomes impossible*,” stated a nurse (P11, female, 8 years’ experience). These tasks, often unrelated to direct patient care, further marginalized PE in daily practice.

### Cultural resistance

Cultural factors, shaped by Hamadan’s local norms and Iran’s hierarchical healthcare culture, created significant barriers to PE, affecting the process of nurse-patient interactions [13]. These barriers stemmed from both patient and nurse attitudes, rooted in sociocultural misconceptions.

- **Lack of patient acceptance of PE**: Patients often perceived nurses as task-oriented rather than educators, influenced by a physician-centric culture. “*Patients think we’re just for injections*,* not teaching*,” said a nurse (P5, female, 15 years’ experience).
- **Nurses’ negative attitudes toward PE**: Some nurses internalized PE as an additional burden rather than a core responsibility. “*Colleagues say PE isn’t our job; it’s extra work*,” noted a nurse (P8, female, 2 years’ experience). This attitude was particularly prevalent among less experienced nurses, who felt overwhelmed by clinical demands.
- **Belief in PE ineffectiveness**: Both nurses and patients doubted PE’s impact, particularly in Hamadan’s traditional context. “*Patients ignore education*,* believing only doctors matter*,” shared a nurse (P1, female, 2 years’ experience). This belief was reinforced by cultural norms prioritizing physician authority.
- **Misunderstandings in nurse-patient communication**: Cultural misconceptions hindered rapport, especially in Hamadan’s diverse population. “*Some patients misinterpret my efforts as unprofessional because I’m not a doctor*,*”* explained a nurse (P9, female, 15 years’ experience).
- **Considering education as difficult**: Nurses perceived PE as a complex task, particularly when addressing diverse patient needs. “*Explaining self-care to patients with different backgrounds is harder than clinical tasks*,” said a nurse (P4, female, 7 years’ experience). This perception was amplified by the lack of culturally tailored educational resources .

### Patient-related limitations

Patient-specific factors, influencing the process and outcomes of PE delivery [13], further complicated nurses’ educational efforts. These barriers were particularly pronounced in Hamadan, where socioeconomic and cultural diversity shaped patient receptivity.

- **Psychological and physical conditions**: Patients’ anxiety, pain, or critical illness reduced their ability to engage with PE. “*Patients are too stressed or sick to listen; they just nod without understanding*,” noted a nurse (P3, male, 22 years’ experience). This was common in acute care settings like emergency or oncology wards.
- **Low literacy and socioeconomic status**: Low health literacy and economic constraints limited patients’ ability to follow educational advice. “*Many patients can’t read or afford to follow advice*,* like buying specific foods for diabetes*,” shared a nurse (P1, female, 2 years’ experience).
- **Language and communication limitations**: Linguistic diversity in Hamadan, including local dialects, posed challenges. “*Some patients speak local dialects we don’t understand*,* so education fails*,” said a nurse (P6, female, 10 years’ experience). This was particularly evident in rural patients, who comprised a significant portion of hospital admissions .
- **Mental preoccupations**: Financial or family concerns distracted patients from engaging with PE. “*They’re worried about costs or family issues*,* not learning about self-care*,” explained a nurse (P5, female, 15 years’ experience).
- **Type of illness**: The nature of patients’ conditions influenced PE acceptance. “*Chronic patients often resist education*,* feeling it’s futile after years of illness*,” noted a nurse (P2, male, 11 years’ experience).

### Nurse-related challenges

Individual nurse factors, rooted in Donabedian’s process domain [13], significantly impeded PE, contributing to “*Culturally silenced care*” by limiting nurses’ ability to engage effectively in educational roles. These barriers interacted with other categories to reinforce PE’s marginalization.

- **Low motivation and emotional exhaustion**: Stress, burnout, and low confidence hindered nurses’ ability to deliver PE. “*Anxiety makes me avoid teaching complex topics*,* like post-surgical care*,” shared a nurse (P10, female, 25 years’ experience).
- **Lack of PE-related knowledge/skills**: Limited training in PE techniques and cultural competency was a common barrier, particularly for novice nurses. “*I wasn’t taught how to educate about stroke care*,* especially for illiterate patients*,” admitted a nurse (P8, female, 2 years’ experience).
- **Inability to manage personal problems**: Personal issues, such as family or financial concerns, distracted nurses. “*My family problems distract me from PE; I can’t focus when I’m worried*,” said a nurse (P9, female, 15 years’ experience).
- **Poor individual and social skills**: Weak communication skills hindered effective PE, especially in culturally diverse settings. “*Some nurses struggle to connect with patients*,* particularly those from rural areas*,” noted a nurse (P6, female, 10 years’ experience). This was compounded by patients’ cultural misconceptions about nurses’ roles .
- **Task prioritization over PE**: Nurses often prioritized clinical tasks over PE due to time constraints. “*When I’m swamped with charting*,* PE feels like an extra burden*,” shared a nurse (P4, female, 7 years’ experience).
- **Role ambiguity**: Uncertainty about nurses’ educational roles further marginalized PE. “*Some colleagues don’t see PE as our responsibility; they leave it to doctors*,” explained a nurse (P7, male, 8 years’ experience).

## Discussion

This study sheds light on the challenges nurses face in providing patient education (PE) in Hamadan’s public hospitals, where hospital policies, cultural attitudes, and limited resources combine to limit their efforts. By framing these barriers within Donabedian’s model of healthcare quality (structure, process, outcome) [13], we uncover how systemic issues and local cultural norms create a cycle where PE is often overlooked, a pattern we describe as “Culturally silenced care.” These findings build on prior research while offering new perspectives for improving nursing practice in Iran and similar healthcare systems worldwide.

### Organizational barriers

Hospital systems in Hamadan often prioritize clinical tasks over PE, leaving nurses with little time or support to educate patients. Unlike earlier studies in Iran that focused on time constraints [7, 8], our findings highlight a unique challenge: mandatory PE policies, meant to ensure accountability, often lead to rushed, superficial sessions. One nurse explained, “We do PE to check a box, not because it’s valued” (P2, male, 11 years’ experience). This issue mirrors challenges in other resource-limited settings, where policy-driven pressures can undermine care quality [6, 18]. To address this, hospitals could reduce nurse-patient ratios and provide dedicated spaces for education, such as private consultation rooms, to make PE a practical part of daily work.

### Cultural challenges

Cultural norms in Hamadan, such as patients’ reliance on family advice or preference for physician-led care, make it harder for nurses to deliver effective PE. Patients often see nurses as task-focused rather than educators, a perception rooted in Iran’s hierarchical healthcare culture [12]. For example, one nurse noted, “Patients think only doctors’ advice matters; they dismiss our teaching” (P5, female, 15 years’ experience). This echoes findings from other culturally diverse settings, where traditional beliefs shape patient expectations [11, 16]. Training nurses in cultural sensitivity, tailored to Hamadan’s mix of urban and rural communities, could help. For instance, involving family members in education sessions or using simple, locally relevant examples (like dietary advice based on common foods) might bridge these gaps.

### Patient-related barriers

Patients’ low health literacy and economic struggles, common in Hamadan, further complicate PE. Many patients, worried about medical costs or family responsibilities, find it hard to focus on learning self-care [9]. “They’re too stressed to listen; they just nod,” shared one nurse (P3, male, 22 years’ experience). Similar challenges appear in other low-resource settings, where tailored education is critical [11]. Hospitals could provide simple tools, like brochures in local dialects or short videos, to make PE more accessible for patients with limited literacy.

### Nurse-related challenges

Nurses’ own challenges, such as limited training or burnout, also hinder PE. Novice nurses, in particular, feel unprepared to educate diverse patients. “I wasn’t taught how to explain stroke care to illiterate patients,” admitted one nurse (P8, female, 2 years’ experience). Yet, some nurses succeeded when given enough time or training, suggesting room for improvement [6, 17]. Regular workshops on PE techniques and cultural competency could boost nurses’ confidence and skills, aligning with global calls for better professional development [19].

### What sets this study apart

Unlike prior research that focused mainly on organizational barriers [6, 18], this study reveals how mandatory PE policies and cultural resistance work together to sideline education. By exploring nurses’ experiences in Hamadan, we show how local factors—like reliance on religious beliefs or linguistic diversity—shape these challenges. This perspective adds a new layer to the literature, especially for hierarchical healthcare systems in countries like Pakistan or Egypt, where similar dynamics exist.

### Practical steps forward

To break the cycle of “Culturally silenced care,” hospitals in Iran and similar settings should take practical steps. First, revising mandatory PE policies to focus on quality rather than compliance could encourage meaningful education. For example, instead of requiring nurses to document every PE session, hospitals could evaluate its impact through patient feedback. Second, creating dedicated spaces for PE, like quiet consultation areas, would help nurses teach without interruptions. Third, training programs tailored to Hamadan’s diverse population—covering local dialects and cultural norms—could improve nurse-patient communication. Finally, using digital tools, such as mobile apps with simple self-care guides, could reach patients with low literacy, a strategy that has worked in other low-resource settings [10].

### Interplay of barriers

Mandatory PE policies, a structural barrier, often clashed with cultural resistance, leading to rushed and ineffective educational sessions. For example, nurses reported that hospital requirements to document PE activities pressured them into superficial compliance, as one nurse stated: “We do PE to check a box, not because it’s valued” (P2, male, 11 years’ experience). This “checkbox exercise” was exacerbated by patients’ cultural misconceptions, rooted in Iran’s hierarchical healthcare culture, where nurses are perceived as task-oriented rather than educators [12]. A nurse noted, “Patients think only doctors’ advice matters; they dismiss our teaching” (P5, female, 15 years’ experience). This dynamic, intensified by Hamadan’s traditional practices—such as reliance on family advice or religious beliefs—created a cycle where nurses’ efforts were devalued, further discouraging meaningful PE [10, 16].

Patient-related limitations, such as low health literacy and socioeconomic constraints, further compounded these challenges. In Hamadan, where diverse populations face economic hardships, patients often prioritized financial concerns over education, as one nurse observed: “They’re worried about costs, not learning about self-care” (P5, female, 15 years’ experience). Linguistic diversity, a prominent feature in Hamadan, added another layer of complexity, with nurses struggling to communicate effectively: “Some patients speak local dialects we don’t understand” (P6, female, 10 years’ experience). These barriers interacted with nurses’ own challenges, such as limited training in PE or cultural competency, which hindered their ability to tailor education to diverse needs [8]. For instance, a nurse admitted, “I wasn’t taught how to educate about stroke care, especially for illiterate patients” (P8, female, 2 years’ experience).

Organizational constraints, including high workloads and inadequate infrastructure, further amplified these issues. Nurses reported that excessive non-nursing tasks, like paperwork, left little time for PE: “Paperwork eats up our energy; PE becomes impossible” (P11, female, 8 years’ experience). The absence of dedicated spaces for education forced nurses to conduct sessions at the bedside, often in chaotic environments, which clashed with patients’ psychological conditions, such as anxiety or preoccupation [9]. This interplay created a vicious cycle where structural deficits limited nurses’ capacity, cultural resistance reduced patient receptivity, and nurses’ lack of training hindered effective delivery, collectively silencing PE [6, 18].

Despite these barriers, variability in practice highlighted potential for improvement. Some nurses overcame constraints when given adequate time or training. For example, a nurse shared, “When I have fewer patients, I can explain diabetes care properly, and patients listen” (P7, male, 8 years’ experience). This suggests that addressing structural barriers, such as reducing nurse-patient ratios, and enhancing professional development through targeted PE training could disrupt this cycle [6, 17]. Moreover, cultural competency training tailored to Hamadan’s linguistic and traditional diversity could bridge communication gaps and align PE with local norms [2]. For instance, incorporating simple, culturally relevant materials or interpreter services could enhance patient engagement, particularly for those with low literacy or linguistic barriers [2, 11].

This interplay underscores the need for holistic interventions that address the interconnected nature of these barriers. By reforming mandatory policies to prioritize quality over compliance, providing dedicated PE spaces, and investing in cultural competency training, hospitals can empower nurses to deliver effective education [9]. These strategies, applicable to other resource-constrained settings with hierarchical healthcare cultures, highlight the global relevance of this study’s findings [11]. Future research should explore patients’ and managers’ perspectives to further elucidate this interplay and develop comprehensive PE frameworks that enhance nursing practice and patient outcomes.

Individual nurse factors significantly impeded patient education (PE), contributing to the overarching theme of “Culturally silenced care” by limiting nurses’ ability to effectively engage in educational roles within Hamadan’s public hospitals. These barriers, rooted in Donabedian’s process domain [13], encompassed psychological factors, inadequate professional knowledge, inability to manage personal problems, and poor individual and social skills that undermine the therapeutic nurse-patient relationship [21]. This interplay of individual challenges, compounded by systemic and cultural constraints, marginalized PE, reducing it to a peripheral task in nursing practice.

These nurse-related challenges did not exist in isolation but interacted with organizational, cultural, and patient-related barriers to reinforce “Culturally silenced care.” For example, inadequate training (nurse-related) compounded the lack of dedicated PE spaces (organizational), forcing nurses to deliver rushed education at the bedside, which clashed with patients’ low health literacy (patient-related) and cultural resistance to nurse-led education (cultural). However, variability in practice offered hope for improvement. Some nurses overcame these barriers when provided with sufficient time or training. “When I took a short course on patient communication, I felt more confident teaching heart failure care” (P7, male, 8 years’ experience). This suggests that targeted interventions, such as continuous professional development and cultural competency training, could enhance nurses’ skills and confidence, aligning with global recommendations for improving PE delivery [6, 17].

These findings align with recent studies from multicultural and resource-limited settings in Saudi Arabia [22] and other middle-income countries [23], which similarly emphasize the combined impact of structural constraints, cultural factors, and organizational barriers on patient education.

### Limitations

This study has several limitations that should be considered when interpreting the findings. First, it was conducted exclusively in public hospitals of Hamadan province, which may limit the transferability of findings to private hospitals, teaching hospitals in large metropolitan cities, or other regions of Iran with different resource levels and cultural contexts.

Second, the study captured only nurses’ perspectives. The absence of patients’ and hospital managers’ viewpoints is an important limitation, as these stakeholders may perceive structural and cultural barriers to patient education differently. Triangulating nurses’ experiences with those of patients and administrators in future studies would provide a more comprehensive understanding of the phenomenon.

Despite these limitations, the use of maximum variation sampling, rich qualitative data, prolonged engagement, and thick description of the context enhance the credibility and potential transferability of the findings to other similar resource-limited, hierarchical healthcare systems in middle-income countries.

### Future research

To build on these findings, future research should adopt a multi-stakeholder approach by exploring the perspectives of patients and hospital managers alongside nurses. Such triangulation would provide a more comprehensive understanding of structural and cultural barriers to patient education within the context of “Culturally silenced care.” Additionally, intervention studies are warranted to test context-specific strategies, such as culturally tailored communication training for nurses, restructuring of mandatory PE policies, provision of dedicated education spaces, and development of low-literacy educational materials, in different Iranian provinces and similar resource-limited settings.

### Cultural influences in Hamadan

In Hamadan, reliance on religious beliefs and family advice often overshadows nurse-led PE, as patients may prioritize traditional practices over medical education [10]. Integrating culturally sensitive approaches, such as involving family members in educational sessions, could enhance patient engagement and align PE with local norms [2].

#### Implications for practice

To overcome “Culturally silenced care,” Iranian hospitals should prioritize PE by allocating dedicated spaces, reducing nurse-patient ratios, and revising mandatory policies to focus on quality over compliance [9, 20]. Cultural competency training, tailored to Hamadan’s linguistic and traditional diversity, is critical [2, 19]. Continuous professional development programs for nurses can further enhance PE delivery [17]. These interventions are applicable to other resource-constrained settings with hierarchical healthcare cultures, enhancing global nursing practice. Future research should explore patients’ and managers’ perspectives to develop holistic PE strategies and validate findings across diverse Iranian contexts. Incorporating digital tools, such as mobile apps for patient education, could enhance accessibility and engagement, particularly for patients with low health literacy [10].

#### Limitations

Conducted in Hamadan’s public hospitals, this study’s findings may not fully generalize to other settings. However, rich descriptions and diverse sampling enhance transferability to similar resource-limited healthcare systems.

## Conclusion

This study reveals that “Culturally silenced care” arises from the interplay of organizational, cultural, patient-related, and nurse-related barriers to PE in Hamadan’s public hospitals. Key findings, including the paradoxical impact of mandatory PE policies, underscore the need for targeted interventions. Hospitals should establish dedicated PE spaces, implement cultural competency training tailored to Iran’s diverse sociocultural context, and reform policies to prioritize quality education. These strategies, applicable to other hierarchical healthcare systems, can empower nurses to deliver effective PE, improving patient outcomes and healthcare equity in resource-limited settings. Future studies should incorporate patients’ and managers’ perspectives to develop comprehensive PE frameworks. The novel finding of mandatory PE policies fostering superficial compliance highlights the need for global healthcare systems to prioritize quality-driven educational reforms.

## Supplementary Information

Below is the link to the electronic supplementary material.

Supplementary Material 1

## Data Availability

The datasets used and analyzed during the current study are available from the corresponding author on reasonable request.

## Abbreviations

PE: Patient Education
