# Supplementary material for: Structural and cultural barriers to patient education from the perspective of Iranian nurses: a qualitative study
Source: BMC Nurs. 2026 Jun 1;25:518. doi: 10.1186/s12912-026-04819-1 (PMC13244940; doi:10.1186/s12912-026-04819-1)
Supplement: Supplementary file 1 — Supplementary Material 1 [file 12912_2026_4819_MOESM1_ESM.docx]

**supplementary file 1**

**Interview Guide**

**Title:** Interview Guide for the Study: “**Barriers to patient education from nurses’ perspectives: a qualitative content analysis**”

**Introduction for Participants**

Thank you for agreeing to participate in this study. The purpose of this interview is to explore your experiences and perceptions regarding the barriers to patient education in your clinical practice.

There are no right or wrong answers; we are genuinely interested in understanding your personal views and professional experiences.

Please be assured that your identity and any personal information will remain completely confidential. Your name or any identifying details will not be published or disclosed at any stage of the research or in any resulting publications.

Participation in this study is entirely voluntary. You have the right to decline to answer any question or to withdraw from the interview and the study at any time without any negative consequences. Your decision to participate or withdraw will have no impact on your job status, performance evaluation, or workplace responsibilities.

The information you share will be used solely for research purposes to improve understanding of the challenges related to patient education among nurses. Your honest and open responses are greatly appreciated.

**Main Questions:**

- Could you please describe your experiences with patient education in your Work shifts in hospital wards?
- Can you recall a situation where patient education was not provided? What were the reasons?
- What factors make it difficult for you to provide patient education? or What factors contribute to PE non-provision?
- In your opinion, what are the main challenges in delivering patient education to hospitalized patients?

**Probing Questions (used as needed):**

- Can you explain more about that?
- Could you give me an example?
- How did you feel in that situation?
- Why do you think that happened?

**Closing Questions:**

- Is there anything else you would like to add about patient education barriers?
- Do you have any suggestions to improve patient education in hospitals?
